# The influence of the R47H triggering receptor expressed on myeloid cells 2 variant on microglial exosome profiles

**DOI:** 10.1093/braincomms/fcab009

**Published:** 2021-02-03

**Authors:** Anna Mallach, Johan Gobom, Henrik Zetterberg, John Hardy, Thomas M Piers, Selina Wray, Jennifer M Pocock

**Affiliations:** 1 Department of Neuroinflammation, Queen Square Institute of Neurology, University College London, London WC1N 1PJ, UK; 2 Department of Psychiatry and Neurochemistry, Institute of Neuroscience and Physiology, University of Gothenburg, S-431 80, Mölndal, Sweden; 3 Clinical Neurochemistry Laboratory, Sahlgrenska University Hospital, S-431 80, Mölndal, Sweden; 4 Department of Neurodegenerative Disease, University College London Queen Square Institute of Neurology, London WC1N 1PJ, UK; 5 Department of Neurodegenerative Disease, University College London Queen Square Institute of Neurology, London WC1N 3BG, UK; 6 UK Dementia Research Institute University College London, London WC1E 6BT, UK; 7 Reta Lila Weston Institute, UCL Queen Square Institute of Neurology, London WC1N 1PJ, UK; 8 NIHR University College London Hospitals Biomedical Research Centre, London, UK; 9 Institute for Advanced Study, The Hong Kong University of Science and Technology, Hong Kong SAR, China

**Keywords:** exosomes, Alzheimer’s disease, microglia, TREM2

## Abstract

Variants in the triggering receptor expressed on myeloid cells 2 gene are linked with an increased risk of dementia, in particular the R47H^het^ triggering receptor expressed on myeloid cells 2 variant is linked to late-onset Alzheimer’s disease. Using human induced pluripotent stem cells-derived microglia, we assessed whether variations in the dynamics of exosome secretion, including their components, from these cells might underlie some of this risk. We found exosome size was not altered between common variant controls and R47H^het^ variants, but the amount and constitution of exosomes secreted were different. Exosome quantities were rescued by incubation with an ATP donor or with lipids via a phosphatidylserine triggering receptor expressed on myeloid cells 2 ligand. Following a lipopolysaccharide or phagocytic cell stimulus, exosomes from common variant and R47H^het^ microglia were found to contain cytokines, chemokines, APOE and triggering receptor expressed on myeloid cells 2. Differences were observed in the expression of CCL22, IL-1β and triggering receptor expressed on myeloid cells 2 between common variant and R47H^het^ derived exosomes. Furthermore unlike common variant-derived exosomes, R47H^het^ exosomes contained additional proteins linked to negative regulation of transcription and metabolic processes. Subsequent addition of exosomes to stressed neurones showed R47H^het^-derived exosomes to be less protective. These data have ramifications for the responses of microglia in Alzheimer’s disease and may point to further targets for therapeutic intervention.

## Teaser

Mallach *et al.* investigated the effect of the R47H *TREM2* variant in human induced pluripotent stem cell microglia-derived cells on exosome secretion and content. They found decreased exosome secretion rate from *TREM2* variants, linked to decreased energy availability and differences in exosomal protein content, which influenced the viability of neuron-like cells.

## Graphical Abstract

**Graphical Abstract:**
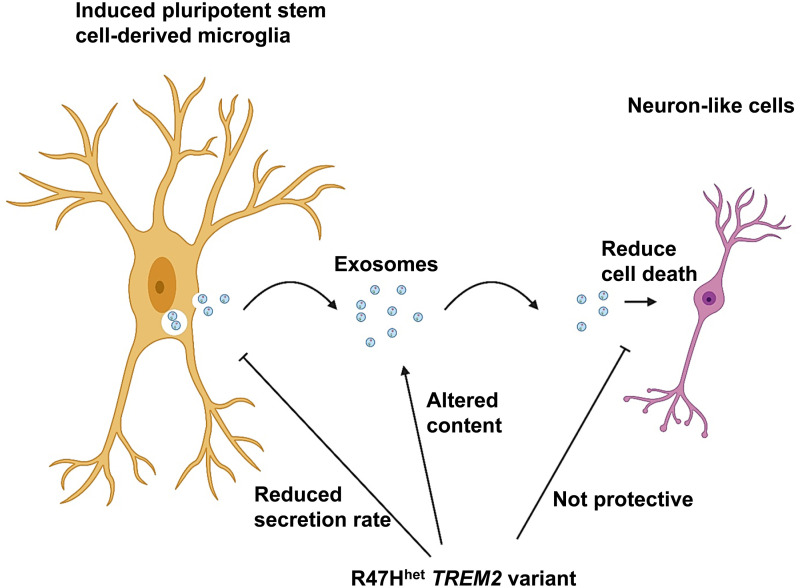

## Introduction

Alzheimer’s disease is the most common neurodegenerative disease with complex underlying disease mechanisms leading to inflammation in the brain and neuronal death (Minagar *et al.*, 2002; Heneka *et al.*, 2013; Zhang et al., 2013). Genome-wide association studies have linked enhanced risk of developing Alzheimer’s disease with inflammation, notably the innate immune cells of the brain, microglia (Naj *et al.*, 2011; Guerreiro *et al.*, 2013; Jonsson *et al.*, 2013). Microglia support neuronal functioning and viability through a range of processes, such as synaptic pruning, production of neurotrophic factors and reacting to threats in the environment (Minagar *et al.*, 2002; Penzes *et al.*, 2011; Parkhurst *et al.*, 2013; Miyamoto *et al.*, 2016). The triggering receptor expressed on myeloid cells-2 (*TREM2*) is involved in this surveillance function, as mutations in the gene for this receptor have been shown to reduce phagocytosis, migration and response to extracellular stimuli (Ulland *et al.*, 2017; Zhong *et al.*, 2017; Garcia-Reitboeck *et al.*, 2018; Piers *et al.*, 2020). Heterozygous mutations in *TREM2*, such as the R47H^het^ mutation, are associated with Alzheimer’s disease (Guerreiro *et al.*, 2013; Jonsson *et al.*, 2013), whilst homozygous missense *TREM2* mutations such as T66M cause Nasu-Hakola disease, a rare early-onset dementia with bone cysts (Paloneva *et al.*, 2002; Guerreiro *et al.*, 2013).

Exosomes are small extracellular vesicles ranging from 30–200 nm in size (Hooper *et al.*, 2012; Yang *et al.*, 2018; Mathieu *et al.*, 2019), created through endocytosis in multi-vesicular bodies and subsequent fusion and release (Hsu *et al.*, 2010). Microglial exosomes have been shown to fulfil different functions, including supporting neuronal viability and functioning (Antonucci *et al.*, 2012; Gupta and Pulliam, 2014; Asai *et al.*, 2015). Upon activation of microglia with the toll-like receptor 4 agonist, lipopolysaccharide (LPS), subsequently secreted exosomes have been shown to contain inflammatory cytokines (Yang *et al.*, 2018) and in turn, differentially affect neighbouring neurons. Since *TREM2* variants and knock-outs have been shown to have an altered responsiveness to pathogenic stimulation (Ito and Hamerman, 2012) and display an impaired metabolism (Ulland *et al.*, 2017; Piers *et al.*, 2020), this could potentially affect exosomal production and release. We hypothesise that microglia with the Alzheimer’s disease-associated R47H^het^*TREM2* variant have an altered exosome secretion and that the exosomes have a differential effect on the viability of neurons. To study this, we used human induced pluripotent stem cells from patients carrying the R47H^het^ mutation, differentiated into functional microglia (iPS-Mg). We analysed the composition of the exosomes and then examined their potential to influence neurones.

## Materials and methods

### iPS generation and iPS-Mg cells

Ethical permission for this study was obtained from the National Hospital of Neurology and Neurosurgery and the Institute of Neurology joint research ethics committee (study reference 09/H0716/64). R47H^het^ fibroblasts were acquired under a material transfer agreement between University College London and University of California Irvine Alzheimer’s Disease Research Centre (Professor M Blurton-Jones). Lines from 3 different patients carrying the R47H variant were used, with 8 clones in total being used. For one patient line, only two clones were included in the analysis due to chromosome abnormalities in the last clone.

Reprogramming of the R47H^het^ fibroblasts was performed as previously described (Xiang *et al.*, 2018), with plasmids obtained from Addgene (#27077, #27078 and #27080). After 7 days *in vitro* nucleofected cultures were transferred to Essential 8 medium (Life Technologies) and colonies were picked after 25–30 days *in vitro*. Karyotype analysis was performed by The Doctors Laboratory (London). The induced pluripotent stem cells were maintained and passaged in Essential 8 medium. Control lines used in this study were: CTRL1 (kindly provided by Dr Selina Wray, UCL Queen Square Institute of Neurology), BIONi010-C (obtained from EBiSC), SFC840 and SBAD03 (obtained from Stembancc) and KOLF2-C1 (obtained from Sanger Institute, available through EBiSC).

Using previously described protocols, iPS-Mg were generated (Xiang *et al.*, 2018). Briefly, embryoid bodies were generated and maintained for 5 days in 100 ng/ml ROCK inhibitor, 50 ng/ml VEGF, 50 ng/ml BMP-4 and 20 ng/ml SCF. The embryoid bodies were collected and transferred to flasks for further differentiation and kept in X-VIVO15 (Lonza) with 100 ng/ml MCSF and 25 ng/ml IL3. After 4–5 weeks of maturation, progenitor cells are collected and plated in iPS-Mg maintenance medium, containing 100 ng/ml IL-34, 25 ng/ml MCSF and 5 ng/ml TGF-β, with the medium being changed every 3–4 days. Two weeks after plating, maturation medium containing 100 ng/ml CX3CL1 and 100 ng/ml CD200 was added to the cells in addition to IL-34, MCSF and TGF-β for another 3 days to achieve final maturation into iPS-Mg, which was confirmed through a custom-designed gene array testing for 28 microglial genes (Xiang *et al.*, 2018).

### SH-SY5Y culture

SH-SY5Y cells (a kind gift from Professor R De Silva, UCL Queen Square Institute of Neurology) were maintained and passaged in DMEM (ThermoFisher) containing 15% foetal bovine serum and penicillin/streptomycin (20 units/ml). The cells were differentiated using a previously established protocol (Encinas *et al.*, 2000). After 5 days of retinoic acid treatment (10 μM), the serum was removed from the medium and 50 ng/ml BDNF (Peprotech) was added for another 7 days. Exosomes were added to the SH-SY5Y neurons after differentiation for 24 h (unless stated differently), with the amount adjusted to total exosomal protein, this was 6 µg/ml.

Apoptotic neurons were generated from undifferentiated SH-SY5Y through heat-shock for 2 h at 45 °C. The effectiveness of this method to induce apoptosis in SH-SY5Y was confirmed through Annexin V FITC (Miltenyi Biotech)/propidium iodide (PI) double-staining as previously shown (Garcia-Reitboeck *et al.*, 2018).

### iPS-Mg exosome collection and extraction

Medium was changed on the iPS-Mg 24 h before the experiment. Following treatment with 100 ng/ml LPS or apoptotic neurons at a ratio of 2:1 apoptotic neurons: microglia for 24 h, the supernatant from the iPS-Mg was collected and centrifuged for 15 min at 300 g. The supernatant was then used for extracting the exosomes.

Exosomes were extracted using an ExoQuick-TC kit (System Biosciences) by centrifugation of the supernatant for 30 min at 3 000 *g* before adding the ExoQuick solution overnight at 4 °C. Following two further centrifugations at 1500 g for 30 min and 5 min, respectively, the pellets from each step were collected. The final exosomal pellet was either resuspended in Dulbecco’s phosphate buffered saline without Ca^2+^/Mg^2+^, pH 7.0–7.3 or RIPA buffer (50 mM Tris-HCl, 150 nM NaCl, 1% SDS, 1% Triton X-100, 1 mM EDTA), freshly supplemented with a protease inhibitor cocktail [Halt protease and phosphatase inhibitor (Thermo Fisher Scientific)], depending on downstream applications.

### Nanoparticle tracking analysis

Exosomes collected from 500 000 iPS-Mg over 24 h collection were diluted in 100 μl PBS and loaded into the recording chamber of a NanoSight LM10 (Malvern Panalytical). The content was recorded twice for 30 sec for each sample. For the human CSF standard (SBI System Biosciences, #EXOP-530A-1), 5.5 µg of exosomes were imaged. Analysis of the amount and size of particles per sample was completed with the nanoparticle tracking analysis (NTA) version 3.2.

### Proteomic analysis

Liquid chromatography mass spectrometry (LC-MS)-based proteomic analysis was performed using the TMT10-plex method for quantification (McAlister *et al.*, 2014). Protein samples were reduced, alkylated and subjected to trypsin digestion using a filter-assisted sample preparation method, similar to a previously described protocol (Ostasiewicz *et al.*, 2010). Three samples per condition, twelve in total, were arranged into two TMT 10-plex sets, each containing two samples of each condition and one reference sample, consisting of a mixture of all samples. To each 240 µl sample, 12 µl 2 M dithiothreitol were added and the samples were then incubated for 30 min at 60 °C. Urea (8 M, 250 µl) was added, and the samples were loaded on molecular-weight cut-off filters (Nanosep, 30 kDa, Pall). Ultrafiltration was performed by centrifugation at 10 000 *g* for 10 min at RT. The flow-through was discarded and the filters were washed twice with 200 µl 8 M Urea, passing the liquid through the filter by centrifugation as above. The filters were then washed with 200 µl 0.5% sodium deoxycholate, 50 mM triethylammonium bicarbonate (digestion buffer). Alkylation of cysteine disulfides was performed by adding 100 µl 18 mM iodoacetamide in digestion buffer to the filters, and incubating for 20 min at RT in darkness. The liquid was removed by centrifugation and the filters were washed twice with 100 µl digestion buffer. Trypsin (Sequencing grade, modified, Promega) in digestion buffer (14 ng/µl, 70 µl) was added to the filters, followed by incubation at 37 °C overnight. An additional 10 µl trypsin aliquot (0.1 µg/µl) was added and the samples were further incubated for another 4 h. Tryptic peptides were collected by centrifugation. TMT 10-plex reagents (8 µg dissolved in 42 µl acetonitrile) were added to the samples followed by incubation for 1 h at room temperature on a shaker. Hydroxylamine (8 µl, 5%) were added and the samples were incubated for 15 min to inactivate excess TMT reagent, after which the individually TMT-labelled samples were pooled into two multiplex samples. The samples were acidified by addition of 10% trifluoroacetic acid to pH < 2 to precipitate the sodium deoxycholate. The samples were centrifuged at 18 000 *g* for 10 min and the supernatants recovered. The TMT labelled peptides were fractionated by reversed-phase chromatography at basic pH. Briefly, the multiplex samples were re-dissolved in 16 µl 2% AcN, 5 mM ammonium hydroxide and loaded on an Ultimate 3000 HPLC system (Thermo Fisher). Offline peptide separation was performed over an XBridge BEH130 C18 3.5 µm, 2.1 mm × 250 mm analytical column at a flow of 100 µl/min, using a linear gradient and collecting 1 fraction/min for 72 min. Collected fractions were then concentrated to 12 fractions, which were dried by vacuum centrifugation.

For LC-MS, samples were dissolved in 6 µl of 0.1% TFA and analysed by LC-MS on nano-HPLC instrument (Ultimate 3000 RSLC nano, Thermo Scientific) fitted with a 75 µm × 2 cm trap column (PepMap C18, Thermo scientific) and a 75 µm × 50 cm C18 column (PepMap C18, Thermo scientific), coupled to a Fusion Tribrid mass spectrometer (Thermo scientific). Following 10 min sample loading onto the trap, separation was performed using the following gradient between 0.1% formic acid (Buffer A) and 84% acetonitrile (Buffer B): *t* = 10 (min), 0% B; *t* = 105, %B = 40; *t* = 112, %B = 99. The mass spectrometer was operated in the positive ion mode, with the following settings: resolution 120 000; m/z range 350–1400; max injection time 50 ms; AGC target 4e5. Data-dependent acquisition was used to record up to 10 consecutive fragment ion spectra (MS2) per full scan spectrum, selecting precursor ions with charge states 2–7 in decreasing order of intensity and using 15 s dynamic exclusion. The isolation window was set to 1.2 m/z. The instrument settings for the MS2 scans were: resolution 50 000; fixed first mass m/z 110; max injection time 86 ms and AGC target 5e4.

The LC-MS data were processed using the software Proteome Discoverer 2.3 (Thermo). Protein identification was performed using the Mascot software (Matrix Science), searching the human subset of SwissProt, with the following settings: cleavage enzyme: trypsin; precursor mass tolerance: 15 ppm; fragment mass tolerance: 0.05 Da; static modifications: TMT10-plex; dynamic modifications: phosphorylation. Percolator scoring was used, and validation was performed using the target-decoy approach, using 1% FDR as cut-off for peptide identification. Protein identifications were accepted that had at least one matching peptide.

### Microglial gene array

A custom gene array based on published microglial expression data (Butovsky *et al.*, 2014; Muffat *et al.*, 2015; Abud *et al.*, 2017; Haenseler *et al.*, 2017) was used to confirm a microglial signature in our iPS-Mg cultures (TaqMan Array Plate 32 plus Candidate Endogenous Control Genes; Thermo Fisher Scientific, see Table 1), as previously described (Xiang *et al.*, 2018). The heatmap was generated through MATLAB.

**Table 1:** Microglial gene signature primer details

Gene name	Primer ID
18 s rRNA	Hs99999901_s1
GAPDH	Hs99999905_m1
HPRT	Hs99999909_m1
GUSB	Hs99999908_m1
APOE	Hs00171168_m1
C1QA	Hs00706358_s1
C1QB	Hs00608019_m1
ITGAM	Hs00167304_m1
CSF1R	Hs00911250_m1
CX3CR1	Hs01922583_s1
GAS6	Hs01090305_m1
GPR34	Hs00271105_s1
AIF1	Hs00610419_g1
MERTK	Hs01031979_m1
OLFML3	Hs01113293_g1
PROS1	Hs00165590_m1
SALL1	Hs01548765_m1
SLCO2B1	Hs01030343_m1
TGFBR1	Hs00610320_m1
TMEM119	Hs01938722_u1
*TREM2*	Hs00219132_m1
BIN1	Hs00184913_m1
CD33	Hs01076282_g1
SPI1	Hs02786711_m1
HEXB	Hs01077594_m1
ITM2B	Hs00222753_m1
C3	Hs00163811_m1
A2M	Hs00929971_m1
C1QC	Hs00757779_m1
RGS1	Hs01023772_m1
FTL	Hs00830226_gH
P2RY12	Hs01881698_s1

#### Immunoblotting

For the detection of exosomal CD63, exosomal lysate was deglycosylated using a PNGase kit (New England Biolabs), following manufacturer’s instructions. For all other exosomal markers, the exosomal lysates were not deglycosylated. Exosomal pellets or iPS-Mg cells were lysed in RIPA buffer as described above. Cell samples were cleared of nuclear material and cell fragments by centrifugation at 15 000 g for 15 min. The cell lysate was denatured using sample buffer and separated by SDS-PAGE. Proteins were transferred onto nitrocellulose membranes and blocked with 5% milk in phosphate buffered saline solution with 1% TWEEN-20 (PBST). For exosomal blots, positive controls consisted of purified exosomes from human serum or Jurkat T lymphocytes (System Biosciences).

Primary antibodies (Table 2) below were incubated with the blots overnight at 4 °C, with the appropriate secondary fluorescently conjugated antibody incubated at 1:5000 for 1 h at RT.

**Table 2:** List of antibodies used

Antibody	Concentration	Diluted in (%)	Company
**CD63**	1:1000	5 milk	Abcam
**ALIX**	1:1000	5 milk	Cell signalling technologies
**CD9**	1:1000	5 milk	System biosciences
**CD81**	1:1000	5 milk	System biosciences
**Calnexin**	1:1000	1 BSA	Santa Cruz
**β-Actin**	1:10 000	1 milk	Thermofisher

### Exosomal uptake

Uptake of exosomes into SH-SY5Y cells was assessed with fluorescence activated cell sorting (FACs). The exosomes were labelled with cell Vybrant DiI (Thermofisher) at a dilution of 1:200 at 37 °C for 1 h. The excess dye was removed using 3 kDa NMWL columns (Amicon), by centrifuging excess dye out for 20 min at 14 000 g. To control for any remaining unbound DiI present after centrifugation, PBS alone was incubated with the dye as well and used as a negative control. The labelled exosomes were then added to SH-SY5Y for 2 h, before the cells were detached using PBS and analysed by FACS. As a negative control, some SH-SY5Y were pre-treated with cytochalasin D (10 μM), chlorpromazine (5 µg/ml) or genistein (200 µM) to inhibit phagocytosis, for 30 min before exposure to labelled exosomes. The data were analysed using Flowing Software 2.

To further visualize exosomal uptake, exosomes were stained with cell Vybrant DiO (Thermofisher) at a diulation of 1:200 at 37 °C for 1 h, before excess dye was removed using 3 kDa NMWL columns (Amicon), as described above. The SH-SY5Y were labelled with BioTracker 555 Orange Cytoplasmic Membrane Dye (Merck Millipore) at 2 µg/ml for 10 min. The cytoplasmic membrane labelling was carried out either before exosome incubation in the 1 h condition or after exosome incubation in the 24 h condition. Nuclei were stained with DAPI before being mounted and imaged with a Zeiss LSM710 confocal microscope.

### Cell death FACs

To analyse the ability of exosomes to modify neuronal death, we treated SH-SY5Y cells with hydrogen peroxide (H_2_O_2,_ 100 μM) for 24 h with or without iPS-Mg exosomes. Subsequently SH-SY5Y cells were labelled with propidium iodide (PI, 5 µg/ml) for 15 min before cell death was analysed by FACs. Controls consisted of unstained cells or cells boiled at 95 °C for 1 h prior to PI labelling. Data were analysed using Flowing Software 2 and plotted as % of cells in H2 region/total number of cells.

### ATP analysis

ATP levels were determined in the iPS-Mg using a commercially available ATP kit (#A22066, Thermo Fisher Scientific). The cells were plated in black walled, glass-bottomed 96-well plates. The iPS-Mg were treated with either apoptotic neurons (2:1 apoptotic neurons: iPS-Mg) or cyclocreatine (10 mM) for 24 h before they were lysed in cell lysis reagent (#1715747, Boehringer Mannheim) and centrifuged at 1000 g for 1 min. Then, 10 µl of sample supernatant or ATP standard was added to 100 µl of the ATP reaction mix, after the background luminescence was read. Luminescence was recorded on a Tecan 10 M plate reader. Data were normalized to ATP standard curves and then to the protein concentration of the sample determined by BCA assay.

### Statistical analysis

The results were represented as mean of at least three separate experiments with internal replicates of at least three ± SEM with a *P* value of 0.05 or below considered statistical significant. The results were analysed using Prism Software version 5. Analysis was performed on pooled control lines and pooled R47H^het^ cell lines.

With regard to the LC-MS data, the relative abundances, normalized to the reference sample, were analysed using MATLAB and tested for normality and the potential influence of the different experimental runs. Following from this quality control, the abundances were normalized to the individual reference sample for each experimental run, accounting for the overall different number of proteins detected in the samples. In addition to this, for further analysis, the abundance ratios were log_10_-transformed to generate a normal distribution, allowing for standard statistical tests to be performed on the data. The results from the data base search were analysed using MATLAB. Analysis of the functional effect of the different proteins was conducted using FunRich and STRING.

**Figure 1:**
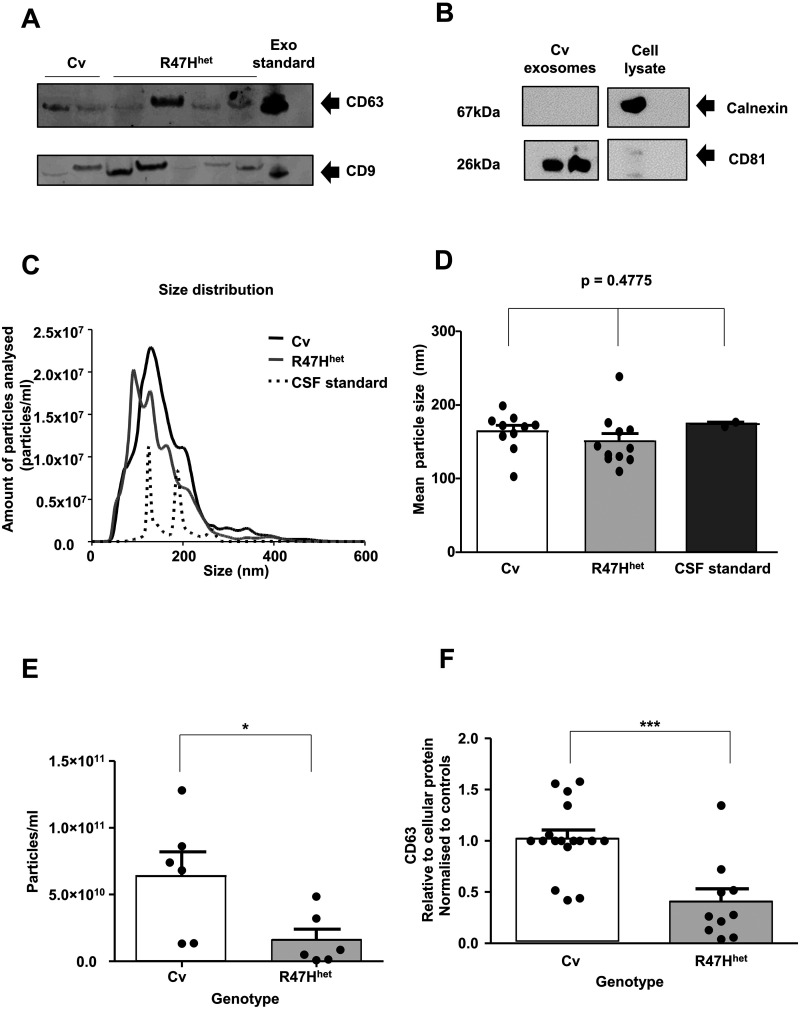
**Exosome characterization.** Western blot of exosomes extracted from Cv and R47H^het^ *TREM2* expressing iPS-Mg express the exosomal markers CD63, and CD9, with exosomes extracted from human serum used as a positive control (Exo standard) in **A**. The extracted exosomal fraction did not contain calnexin, indicating that the fraction is clear of ER contamination, whilst the exosome fraction contained another exosomal marker CD81 in **B**. Size distribution curves of exosomes from Cv and R47H^het^ iPS-Mg, against a human CSF standard assessed by NTA in **C** and **D**. Quantification of the exosome concentration of Cv or R47H^het^ iPS-Mg derived exosomes following NTA in **E** or by western blot of CD63 expression in **F**. The full blots for **A** and **B** can be found in Supplementary Fig. S5 and expanded datasets for **D**–**F** in Supplementary Fig. S4. All data are the mean ± SEM, *n* ≥ 4 with one-way ANOVA in **D** and Students *t*-test in **E** and **F** * *P* < 0.05, *** *P* < 0.001

Power calculations for exosomal size differences as measured through NTA (Fig. 1D) were performed in line with Yang *et al.* (2018) and statistics were performed using the D90 measurement (calculated = 9). We include *n* = 10 for the common variant (Cv) and *n* = 11 for the *TREM2* R47H^het^ and therefore achieve the necessary power required to state that based on the D90 measurement no difference is observed.

## Data availability

All data are available upon request. All data points are shown on graphs.

## Results

### Exosome characterization

To confirm that the particles in the exosomal fraction were indeed exosomes, we assessed whether they displayed common exosomal characteristics. Classical exosomal markers, such as CD63, CD9, ALIX, CD81, HSP70, TSG101 and Flotillin-1 (Hooper *et al.*, 2012; Witwer *et al.*, 2013; Fröhlich *et al*., 2014; Mehdiani *et al.*, 2015; Schiera *et al.*, 2015; Horibe *et al.*, 2018; Chen *et al.*, 2019) were all found to be expressed in the iPS-Mg exosomal fraction of both Cv and R47H^het^ derived exosomes (Fig. 1A and Supplementary Fig. S1C). Calnexin, an endoplasmic reticulum (ER) marker, was absent from the exosomal fraction, but shown to be present in the cell lysate (Fig. 1B) confirming the absence of apoptotic bodies from the exosomal fraction (Lötvall *et al.*, 2014).

The size distribution of the particles as measured through NTA was similar in both Cv and R47H^het^ variants and comparative in size with a human CSF exosome standard (Fig. 1C and D). The average size of the particles was not significantly differently from each other (Fig. 1D). This was further confirmed by the D90 measurement, again showing no statistical size difference between the Cv and R47H^het^ exosomes (Supplementary Fig. S1B). These results confirm that the particles found in the iPS-Mg exosomal fraction are indeed exosomal and that they can be extracted reliably from iPS-Mg with *TREM2* variants.

### Exosome secretion

As exosome secretion is dependent on interactions with the cytoskeleton and energy availability within the cells (Hoshino *et al.*, 2013; Zou *et al.*, 2019), we investigated the exosome secretion concentration in iPS-Mg carrying the R47H^het^ *TREM2* variant, as it has been shown that these cells have deficits in both their cytoskeleton and metabolic fitness (Takahashi *et al.*, 2005; Ulland *et al.*, 2017; Piers *et al.*, 2020). Measurements by NTA indicated that exosomes were enriched following purification (Video 1) but not present in cell medium alone (Video 2). Further NTA measurements indicated that the concentration of exosomal particles secreted over 24 h from the R47H^het^ expressing iPS-Mg compared with Cv was significantly different (Fig. 1E). Subsequent analysis of exosomal CD63, a classical marker of exosomes and an approximation of exosomal number, was significantly decreased (Fig. 1F).

As extracellular stimuli, such as inflammatory signals, can influence the profile of secreted exosomes, both in their size and amount (Qu *et al.*, 2007; Yang *et al.*, 2018), we investigated the amount of exosome secretion following activation of iPS-Mg with 100 ng/ml LPS for 24 h. As we found deficits in exosome secretion in the *TREM2* variant (Fig. 1F), we also activated the *TREM2*-DAP12 pathway through the addition of apoptotic neurons, which express the *TREM2* ligand phosphatidylserine (PS^+^) on their surface (Wang *et al.*, 2015; Garcia-Reitboeck *et al.*, 2018; Shirotani *et al.*, 2019) and used exosomal CD63 as a readout of exosomal production.

**Figure 2:**
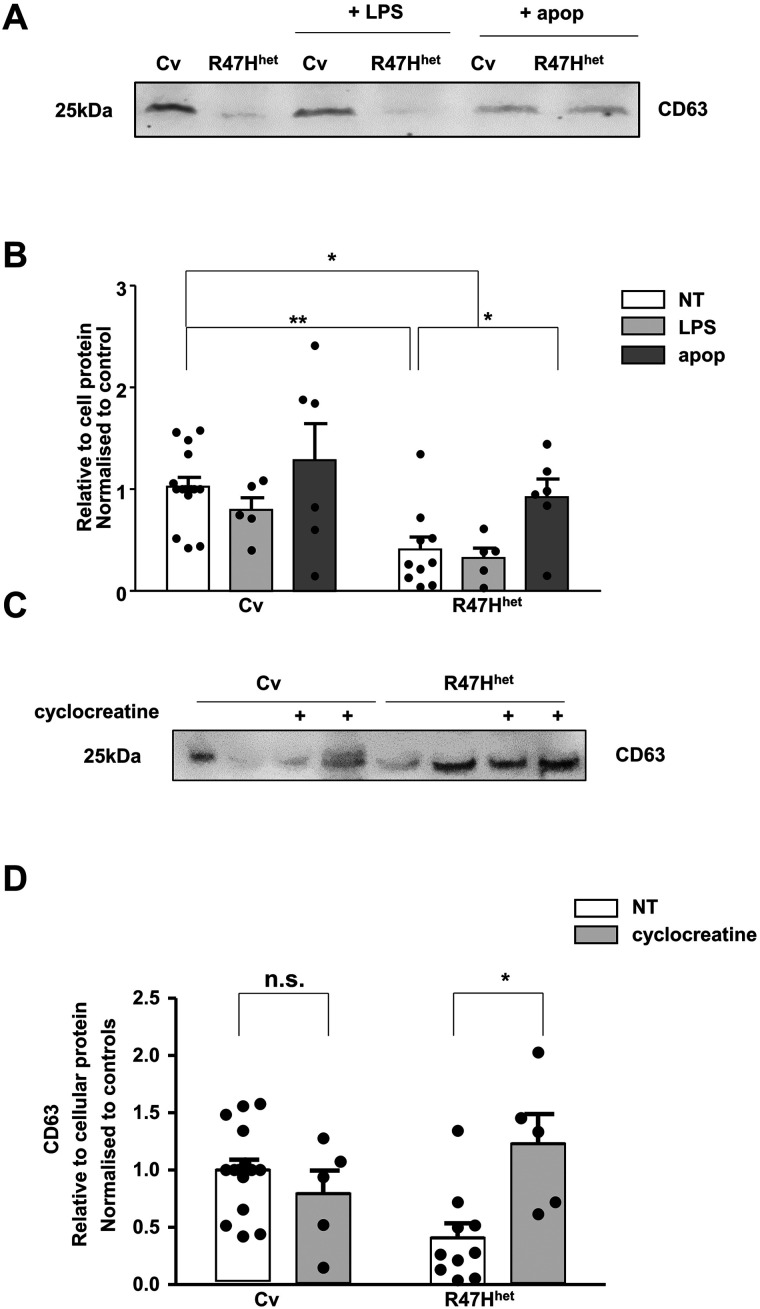
**Exosome secretion can be rescued.** Western blot of exosomal CD63 levels, (used to approximate exosome secretion), following stimulation of iPS-Mg with 100 ng/ml LPS or apoptotic neurons (ratio of 2 apoptotic neurons: 1 iPS-Mg) for 24 h in **A** and quantification of CD63 expression in **B**. Western blot of exosomal CD63 in **C** and CD63 quantification in **D** following exposure of iPS-Mg to 10 mM cyclocreatine for 24 h. The full blots for **A** and **C** can be found in Supplementary Fig. S5 and expanded datasets for **B** and **D** in Supplementary Fig. S4. Data are the mean ± SEM, *n* ≥ 3 following two-way ANOVA followed by Tukey’s post-hoc tests, n.s not significant, * *P* < 0.05, ** *P* < 0.01

CD63 expression was unchanged in exosomes from Cv or R47H^het^ iPS-Mg with LPS or apoptotic neuron treatment (but was significantly lower in unstimulated and LPS stimulated R47H^het^-derived exosomes Fig. 2A and B). Apoptotic neuron-treatment increased exosome secretion from R47H^het^ iPS-Mg (Fig. 2A and B), whilst Cv exosome secretion was not affected. Apoptotic neurons have been shown to supply iPS-Mg with additional energy (Cosker *et al.*, 2020), possibly by providing additional energy via lipid catabolism (Nadjar, 2018), rescuing the energy deficit of R47H^het^ iPS-Mg previously reported (Piers *et al.*, 2020). Based on this, we tested whether the increase in exosome secretion from R47H^het^ following exposure to apoptotic neurons was due to an increase in energy supply. Thus, iPS-Mg were treated with 10 mM cyclocreatine for 24 h, which acts as an ATP supply fuelling oxidative phosphorylation rather than glycolysis, which we have shown to be deficient in *TREM2* variant iPS-Mg (Piers *et al.*, 2020). Again, an increase in exosomal CD63 expression was observed in the R47H^het^ exosomes (Fig. 2C and D), whilst there was no significant difference in Cv, suggesting that the reported energy deficit also underlies the reduced secretion of exosomes in the R47H^het^ iPS-Mg. Furthermore analyses of ATP content revealed that there was a significantly lower level of ATP in R47H^het^ cells relative to Cv, and this was increased to Cv levels by prior exposure to PS^+^ cells or cyclocreatine (Supplementary Fig. S2A).

### Exosome content by MS analysis

**Figure 3:**
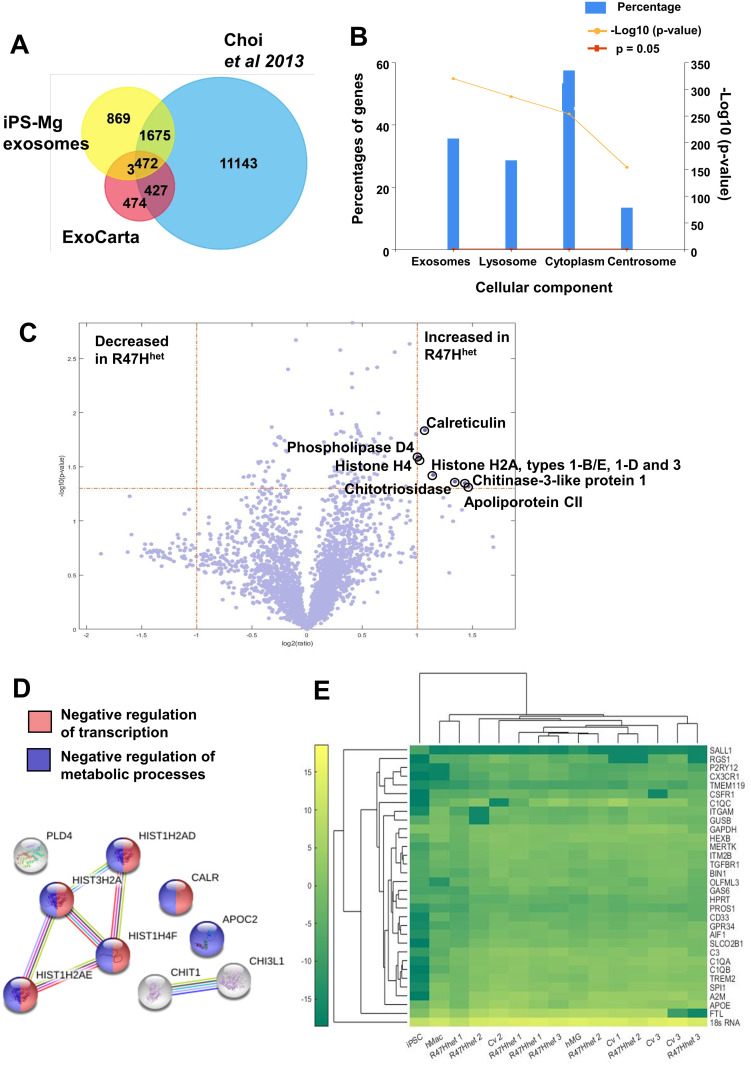
**Proteomics of exosomes.** Mass spectrometry was performed on exosomes extracted from iPS-Mg. Over 70% of the proteins detected by mass spectrometry (blue) have been previously linked to exosomal proteins in **A**. Of about 2147 proteins have been previously published in a meta-analysis of high-throughput proteomics of exosomes (Choi *et al.*, 2013), whilst 475 proteins were shared with the online repository of exosomal proteins (ExoCarta-Accession, red). This was also verified following analysis of the proteins for their cellular compartment of origin in **B**. The proteins are significantly associated with exosomes, followed by the lysosomes and cytoplasm relative to the annotated control genome. This confirms the extracted particles to be exosomes. The difference between exosomes secreted from Cv and R47H^het^ iPS-Mg was compared by volcano plot in **C**. The ratio in abundance of proteins in R47H exosomes, compared with Cv exosomes, was plotted in the *x*-axis whilst the significance of the change, in form of the *P*-value, was plotted in the *y*-axis, meaning that any proteins in the top left and right section had a significant 2-fold change. Nine proteins were found to be significantly packaged into exosomes originating from R47H^het^ iPS-Mg compared with exosomes from Cv iPS-Mg in **C**. The function of these proteins was analysed through STRING analysis in **D**, which indicated that some of the proteins increasingly packaged into R47H^het^ exosomes are involved in the negative regulation of transcription and metabolic processes, *n* = 3 independent experiments. Heatmap of the analysis of microglial gene signature in all iPS-Mg lines and clones used in this study using a custom gene array in **E**

LC-MS performed on exosomes extracted from iPS-Mg revealed over 70% of the proteins detected have been previously linked to exosomal proteins (Fig. 3A) and furthermore, by virtue of their cellular compartment of origin, these proteins were found in exosomes**, (**Fig. 3B**),** providing additional support that the extracted particles from iPS-Mg were exosomes.

Analysis of baseline differences between exosomes secreted from Cv and R47H^het^ iPS-Mg by volcano plot revealed nine proteins with higher abundance in R47H^het^ iPS-Mg (ratio > 2, *P* < 0.05). (Fig. 3C and Supplementary Fig. S2C). STRING analyses revealed that some of these proteins are involved in the negative regulation of transcription and metabolic processes (Fig. 3D).

Analysis of microglial gene signature was assessed in all lines studied using a custom gene array based on previously published expression data (Xiang *et al.*, 2018). Lines from both *TREM2* genotypes used in this study cluster strongly together with only one outlier observed clustering more closely with human macrophages (Fig. 3E). These data suggest differences observed in exosomal content between genotypes is not due to gross microglial gene signature differences at basal.

### Exosome content following LPS or apoptotic cell stimulation

**Figure 4:**
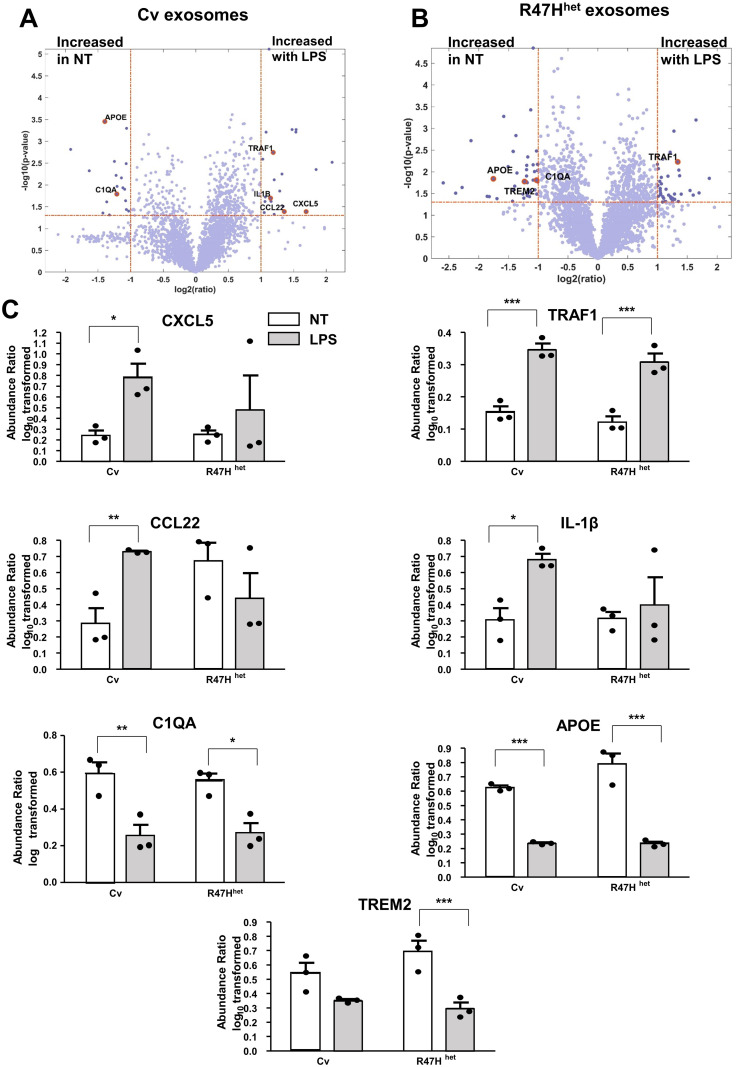
**Stimulation-induced differential changes in exosomal proteins following TLR4 stimulation.** Volcano plots of the effect of LPS stimulation on exosomal proteins in Cv iPS-Mg in **A** and R47H^het^ iPS-Mg in **B**. Proteins of interest were circled and further plotted in **C**. Data are mean ± SEM with *n* = 3 independent experiments. Two-way ANOVA followed by Tukey’s post-hoc test with * *P* < 0.05, ** *P* < 0.01

We investigated changes in the relative expression of exosomal proteins following treatment of iPS-Mg with LPS, as previous research has suggested changes in exosomal proteins occur following activation of microglia (Yang *et al.*, 2018). Volcano plots of proteomic data showed that several proteins differ in abundance in exosomes derived from Cv iPS-Mg and R47H^het^ iPS-Mg with and without stimulation with LPS (Fig. 4A and B, respectively). Following activation with LPS, the content of the iPS-Mg exosomes changed significantly to include increased secretion of cytokines in both Cv and R47H^het^ *TREM2* variants, such as TRAF1, (Fig. 4C), whilst others such as C1QA and APOE showed a significant reduction in exosomes from LPS-treated Cv and R47H^het^ iPS-Mg. *TREM2* was not significantly reduced in exosomes from Cv but was from R47H^het^ exosomes (Fig. 4C).

**Figure 5:**
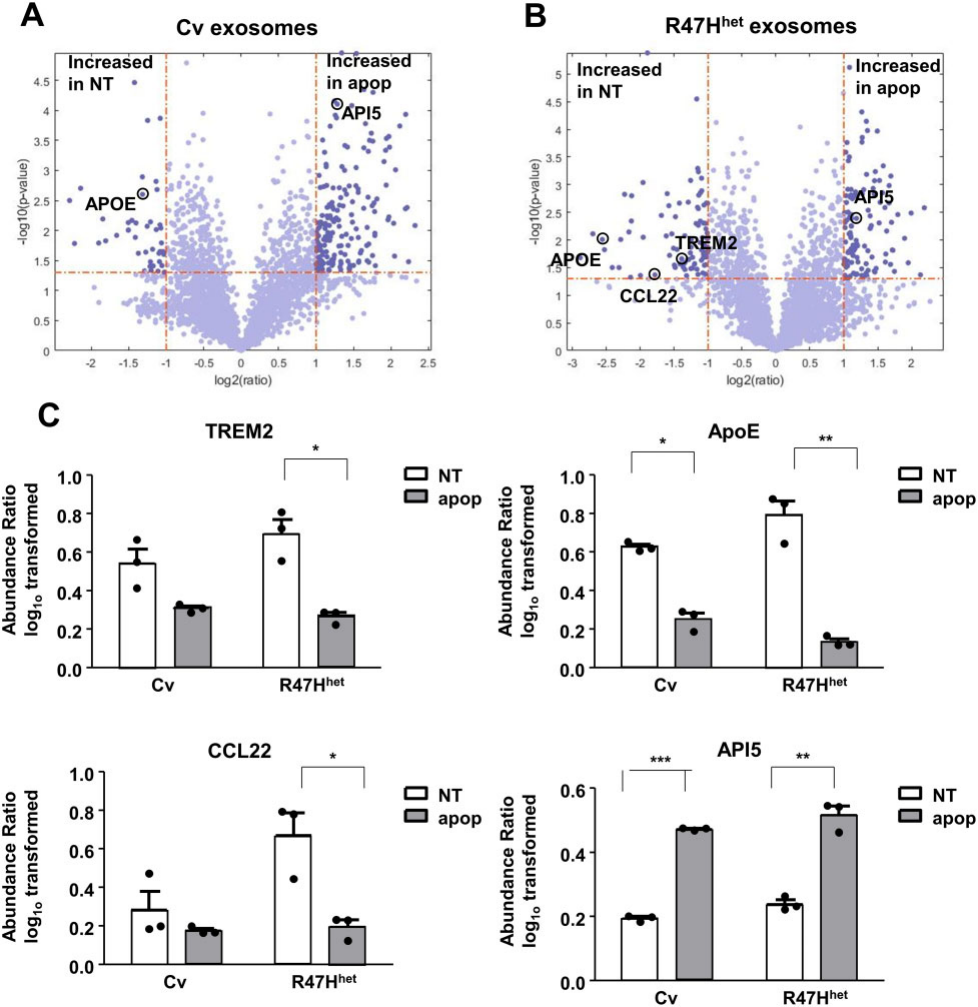
**Changes in exosomal protein following *TREM2* ligand exposure.** Volcano plots for the effect of apoptotic neuron treatment on exosomal proteins in Cv iPS-Mg in **A** and R47H^het^ iPS-Mg in **B**. Proteins of interest were further plotted in **C**. Data are mean ± SEM with *n* = 3 independent experiments. Two-way ANOVA followed by Tukey’s post-hoc test with * *P* < 0.05, ** *P* < 0.01

**Figure 6:**
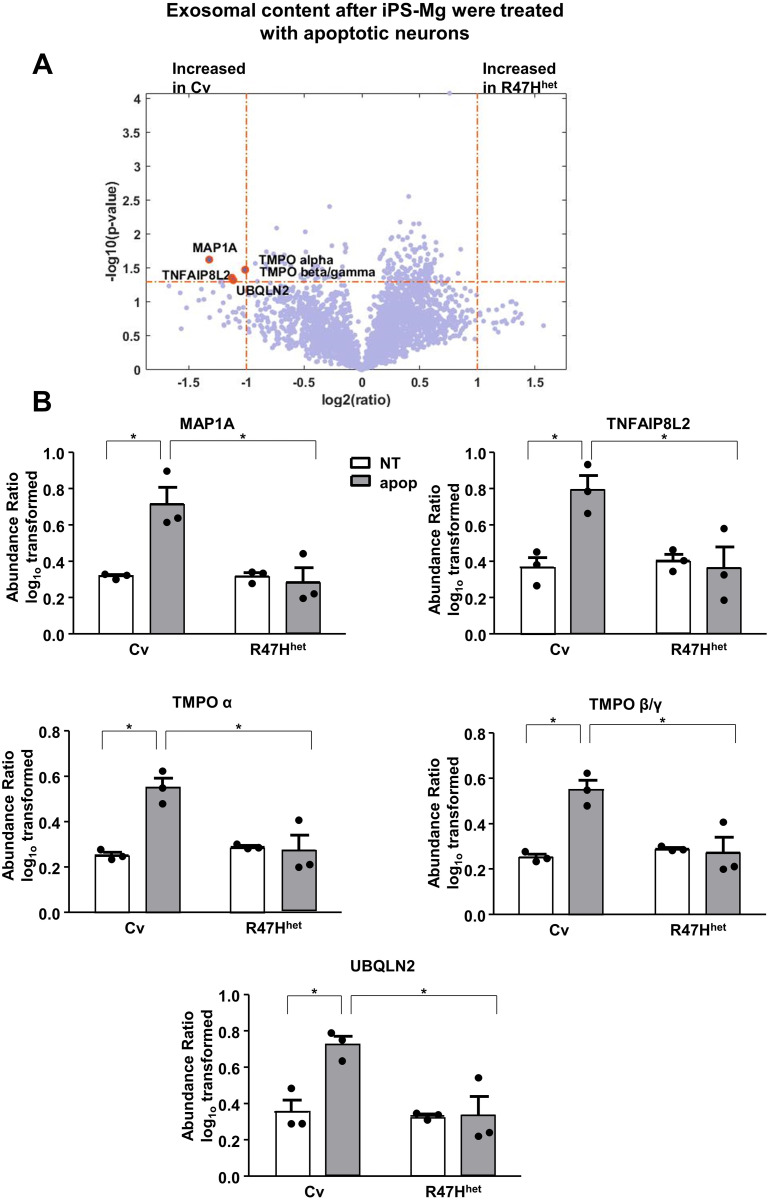
**Comparison of exosomal proteins increased following exposure to a *TREM2* ligand.** Volcano plots comparing the increased abundance levels of exosomal proteins from Cv or R47H^het^ iPS-Mg treated with apoptotic neurons to stimulate *TREM2*-mediated pathways in **A**. Abundance levels of some of the proteins observed to change following this stimulation in **B**. Data are mean ± SEM with *n* = 3. Two-way ANOVA followed by Tukey’s post-hoc test with * *P* < 0.05, ** *P* < 0.01

Volcano plots of proteomic data from Cv or R47H^het^ derived exosomes exposed to apoptotic neurons revealed differences in abundance (Fig. 5A and B). Furthermore CCL22 was significantly reduced in R47H^het^ exosomes compared with Cv following stimulation with apoptotic neurons (Fig. 5C). Furthermore a significant number of proteins were not increased in R47H^het^ derived exosomes compared with Cv following exposure of iPS-Mg to apoptotic neurons (Fig. 6).

### Uptake of iPS-Mg exosomes into neurons

**Figure 7:**
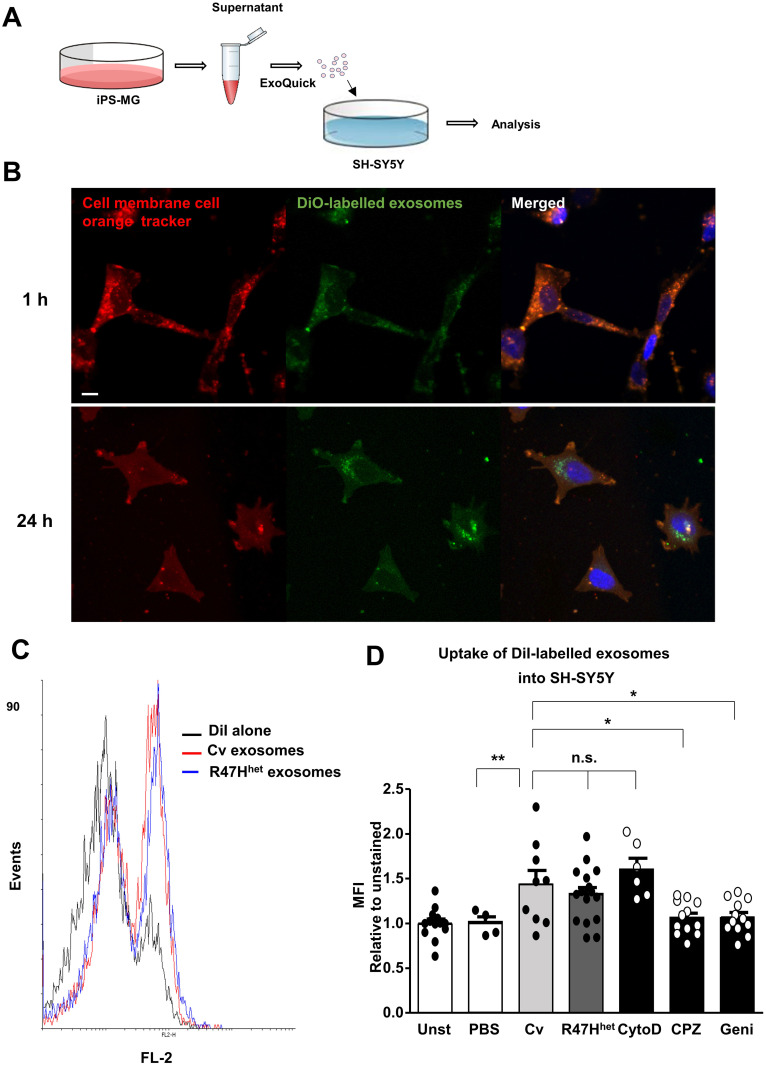
**Uptake of iPS-Mg exosomes into SH-SY5Y.** IPS-Mg derived exosomes were added to differentiated SH-SY5Y (workflow: **A**). Uptake of exosomes into SH-SY5Y was determined by imaging DiO-labelled exosomes inside SH-SY5Y, (the latter were stained with BioTracker 555 Orange Cytoplasmic Membrane Dye, 2 μg/ml) after 1 h and 24 h of incubation with the exosomes in **B** Scale bar = 10 μm. Uptake was quantified by FACs analysis of DiI-labelled exosomes and compared with unstained cells (Unst) in **C** or with Dil-labelled PBS as a negative control (PBS) or following 30 min preincubation with cytochalasin D (10 μM), chlorpromazine (chlorpromazine, 5 µg/ml) or genistein (200 µM) in **D**. Expanded dataset for **D** can be found in Supplementary Fig. S4. Data are the mean ±SEM with one-way ANOVA followed by Tukey’s post-hoc test, *n* ≥ 4. ** *P* < 0.01, * *P* < 0.05, ns, not significant

As microglia exosomes have been shown to affect neuronal functioning (Antonucci *et al.*, 2012; Chen *et al.*, 2019) we determined whether iPS-Mg-derived exosomes from Cv or R47H^het^ cells were taken up into neurons (Fig. 7). Uptake of the exosomes was verified both through confocal microscopy, showing DiO labelled exosomal particles inside the neurons within 1 h of incubation and 24 h (Fig. 7B). This was also quantified by FACs, showing a significant uptake of Dil labelled exosomes into the neurons after 2 h in comparison with unstained cells (Fig. 7C and D). No significant difference was observed in the level of uptake of exosomes from Cv or R47H^het^ iPS-Mg, albeit that the R47H^het^ exosomes appeared to be taken up at slightly lower levels. As a negative control, exosome-free saline solution was incubated with label to control for any carry-over of Vybrant dye, however this appeared not to be a confounding factor (Fig. 7D). Uptake could be inhibited by chlorpromazine or genistein, but not cytochalasin D (Fig. 6Cii).

**Figure 8:**
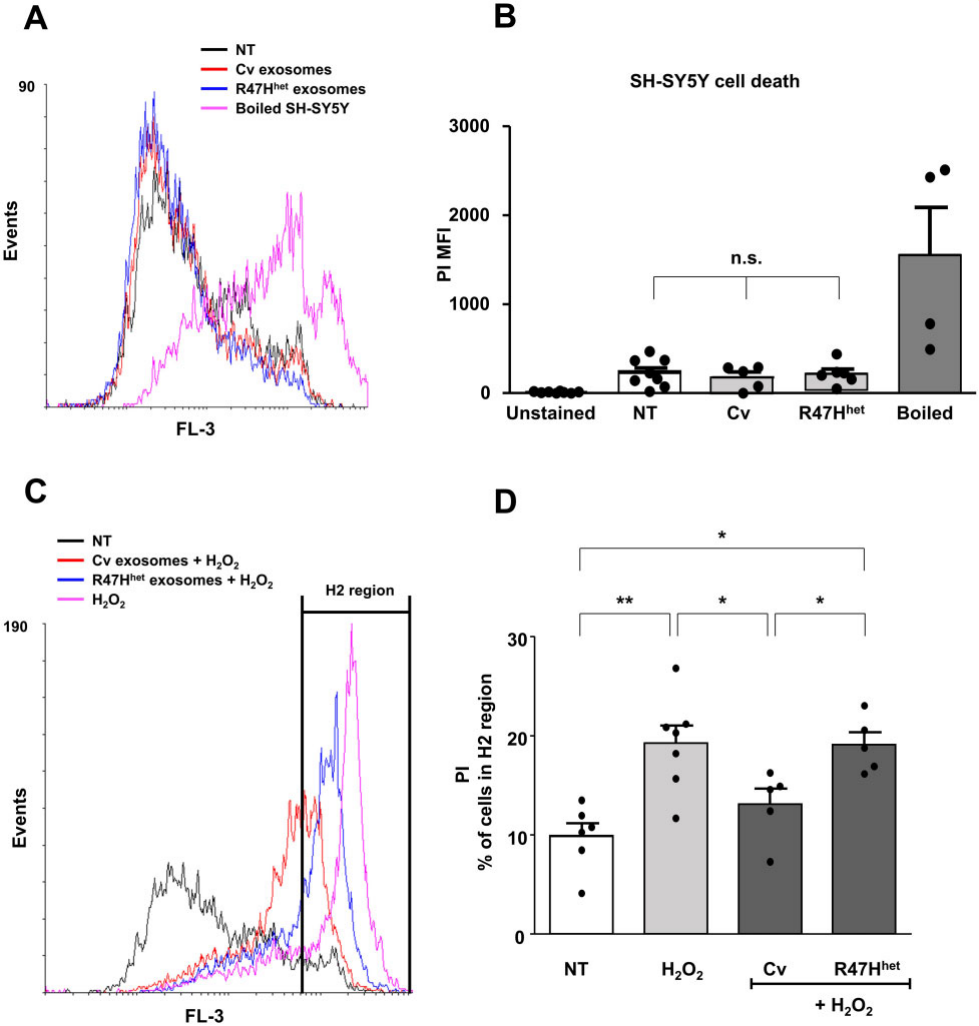
**SH-SY5Y viability following exosome incubation.** The effect of exosomes on the viability of SH-SY5Y assessed with prodidium iodide (PI) staining and FACS analysis. Baseline cell death following addition of exosomes from unstimulated iPS-Mg from Cv or R47H^het^ to SH-SY5Y in **A** and **B**. Cell death in SH-SY5Y challenged with H_2_O_2_ (100 μM) for 24 h in the presence of exosomes from Cv or R47H^het^ iPS-Mg or in **C** and **D**. Expanded datasets for **B** and **D** can be found in Supplementary Fig. S4. One-way ANOVA followed by Tukey’s post-hoc test, with *n* ≥ 5 and *n* ≥ 4 for **B** and **D,** respectively, ** *P* < 0.01

Based on our finding of changes in exosome content in Cv and R47H^het^ exosomes (Figs. 4–6), the effect of exosomes from either Cv or R47H^het^ iPS-Mg on neuronal viability was assessed. Following incubation of exosomes with differentiated SH-SY5Y (see Supplementary Fig. S3 for SH-SY5Y differentiation), no significant differences in the levels of neuronal death were observed after addition of Cv or R47H^het^ exosomes derived from non-treated iPS-Mg (Fig. 8A and B).

Previous research has shown that the protective functions of glial exosomes are more easily revealed when the SH-SY5Y were subjected to H_2_O_2_-induced cell stress (Pascua-Maestro *et al.*, 2019). Based on this, the ability of exosomes from iPS-Mg to rescue SH-SY5Y neurons from H_2_O_2_-induced cell death was investigated (Fig. 8C and D). H_2_O_2_ increased neuronal death significantly (Fig. 8D). However, the addition of Cv iPS-Mg-derived exosomes protected SH-SY5Y neurons from H_2_O_2_-induced death as there was no significant difference between the levels of death in non-treated neurons compared with those treated with Cv exosomes and H_2_O_2_ indicating a protective effect of Cv-derived exosomes (Fig. 8D). Conversely, the level of death in neurons treated with H_2_O_2_ plus exosomes from R47H^het^ iPS-Mg was no different from neurons exposed solely to H_2_O_2_, indicating that these exosomes were not protective (Fig. 8D).

## Discussion

We were able to reliably extract exosomes from iPS-Mg, the exosomes showed a range of common exosomal markers and were also devoid of markers which might indicate contamination from different cellular components, such as ER (Fig. 1B). The average size of the exosomes from cells with or without the R47H^het^ mutation was approximately 150 nm, and a comparison of the size of exosomes from Cv with R47H^het^ indicated that they were not significantly different (Fig. 1D and Supplementary Fig. S1B), suggesting that the R47H^het^ variant does not affect exosome size. Our findings also suggest that the particles as analysed by NTA were potentially larger than those reported elsewhere (Witwer *et al.*, 2013), but in part this can be explained by the measuring technique we used, as even the CSF exosome standard was measured to be around the same size as the iPS-Mg derived exosomes (Fig. 1D). Due to the size of the NTA chamber, during imaging, many particles are out of focus and could skew the average size measured. The R47H^het^ variant appeared to influence exosome number (Fig. 1E), which was also reflected in the decrease in exosomal CD63 (Fig. 1F). This could be due to the metabolic deficit in microglia with this variant (Ulland *et al.*, 2017; Piers *et al.*, 2020), as supplying the cells with additional lipids in the form of apoptotic cells was able to alter this to control levels for the protein we tested as indeed did cyclocreatine, generating ATP in the cells (Kurosawa *et al.*, 2012).

In addition to influencing exosome secretion, the R47H^het^ variant altered the packaging of proteins into exosomes, as shown through proteomic analysis with nine proteins increasingly packaged into exosomes from R47H^het^ iPS-Mg which were found to be involved in negative regulation of metabolic processes (Fig. 3C and D and Supplementary Fig. S2B). In a recent study, one of the most altered Alzheimer’s disease protein co-expression modules was linked to sugar metabolism in glia such as microglia (Johnson *et al.*, 2020). Our data suggest that exosome signalling for metabolism is thus likely to be decreased in R47H^het^ iPS-Mg.

Whether these proteins are present in R47H^het^ exosomes because of the metabolic deficits present in the microglia (Ulland *et al.*, 2017; Piers *et al.*, 2020) is not yet known. However, our study confirms previous research that exosomes from LPS-activated microglia contain more inflammatory cytokines (Yang *et al.*, 2018), although we also found that *TREM2* was present in exosomes but also decreased following LPS stimulation, and significantly so in R47H^het^ derived exosomes (Fig. 4C). There were also differences in the proteins present in exosomes from Cv versus R47H^het^ iPS-Mg, and in particular, a number of proteins were not increased in the latter after exposure to apoptotic cells. This suggests that whilst some proteins can be increased by a *TREM2* ligand in Cv, in the R47H^het^ iPS-Mg, this is not the case.

Whilst full transcriptomic analysis of the iPS-Mg lines used in this study may go further to identify if there were significant differences in the underlying *TREM2* genotypes that could contribute to exosomal content variance, the microglial gene signature array performed here suggests limited differences between the genotypes (Fig. 3E). Strong clustering between all the lines was observed (with the exception of one potential outlier repeat), suggesting that the *TREM2* genotypes do not differ significantly at the microglial signature level. This observation means differences in exosomal content were not likely to be due to differences in the underlying basal microglia signature of the *TREM2* variants.

Exosomes can be taken up through a range of different routes into recipient cells (Mulcahy *et al.*, 2014; Schneider *et al.*, 2017; Horibe *et al.*, 2018). We did not find exosome uptake into SH-SY5Y neurons to be inhibited by Cytochalasin D, which mainly inhibits phagocytosis and macropinocytosis (Kuhn *et al.*, 2014) through inhibition of actin polymerization, but other uptake mechanisms into SH-SY5Y cells such as membrane fusion and lipid raft-mediated endocytosis are still available for the exosomal cell entry, possibly explaining why exosomal uptake into SH-SY5Y was not be blocked. Chlorpromazine, an inhibitor of clathrin-mediated endocytosis (Liu *et al.*, 2007; Kuhn *et al.*, 2014; Wesén *et al.*, 2017) or genistein, an inhibitor of caveolin-mediated endocytosis (Horibe *et al.*, 2018), did inhibit uptake, however.

Since the same amount of exosomal protein from Cv or R47H^het^ iPS-Mg was added to the neurons and no significant differences were observed in uptake, this suggests that the actual composition and content of R47H^het^ exosomes influences neuronal functioning. When we compared whether the exosomes from either Cv or R47H^het^ affected baseline neuronal viability (Fig. 8A and B), there was no difference, suggesting that the composition of exosomes from unstimulated iPS-Mg does not in itself influence neuronal viability over the time course of our experiments. However exosomes from Cv were protective against an H_2_O_2_ stress whilst those from R47H^het^ iPS-Mg were not (Fig. 8C and D); this may be due to our finding of negative regulators of metabolism being preferentially packaged into R47H^het^ exosomes. It was interesting that exosomes contained *TREM2*, present in exosomes from Cv and R47H iPS-Mg, although we do not know as yet if this is the full length protein. It has recently been reported that *TREM2* is present in exosomes in serum and that these exosomes may transport *TREM2* into the brain (Raha-Chowdhury *et al*., 2018). We show here that exosomes were taken up in neurons, the ramifications of which would need to be further investigated.

In this study, we have shown that exosomes can be reliably extracted from human iPS-Mg expressing *TREM2* variants and that disease-associated *TREM2* variants secrete less exosomes compared with Cv, potentially due to the reported metabolic deficits. Differences in exosomal protein content between the Cv and R47H^het^ iPS-Mg may influence the ability of neurons to protect themselves against H_2_O_2_ induced stress or other stresses. These differences may influence the ability of human microglia to protect neurons in Alzheimer’s disease if the microglia express the R47H^het^ variant.

## Supplementary material

Supplementary material is available at *Brain Communications* online.

## Supplementary Material

fcab009_Supplementary_Data

## Notes

See Kronenberg-Versteeg and Varga (https://doi.org/10.1093/braincomms/fcab041) for a scientific commentary on this article.

## Abbreviations

AD =: Alzheimer’s disease
CPZ =: chlorpromazine
Cv =: common variant
CytoD =: Cytochalasin D
DIV =: days *in vitro*
EB =: embryoid bodies
ER =: endoplasmic reticulum
FACs =: fluorescence activated cell sorting
H_2_O_2_ =: hydrogen peroxide
iPSC =: induced pluripotent stem cells
iPS-Mg =: induced pluripotent stem cell-derived microglia
LC-MS =: liquid chromatography mass spectrometry
LPS =: lipopolysaccharide
NTA =: nanoparticle tracking analysis
PBST =: phosphate buffered saline solution with 1% TWEEN-20
PI =: propidium iodide
PS^+^ =: phosphatidylserine
RA =: retinoic acid
SDC =: sodium deoxycholate
SN =: supernatant
TREM2 =: triggering receptor expressed on myeloid cells 2
