# Supplementary material for: The influence of the R47H triggering receptor expressed on myeloid cells 2 variant on microglial exosome profiles
Source: Brain Commun. 2021 Feb 3;3(2):fcab009. doi: 10.1093/braincomms/fcab009 (PMC8244649; doi:10.1093/braincomms/fcab009)
Supplement: fcab009_Supplementary_Data [file fcab009_Supplementary_Data.zip › Supplementary_material.pdf]

Supplementary Figure 1

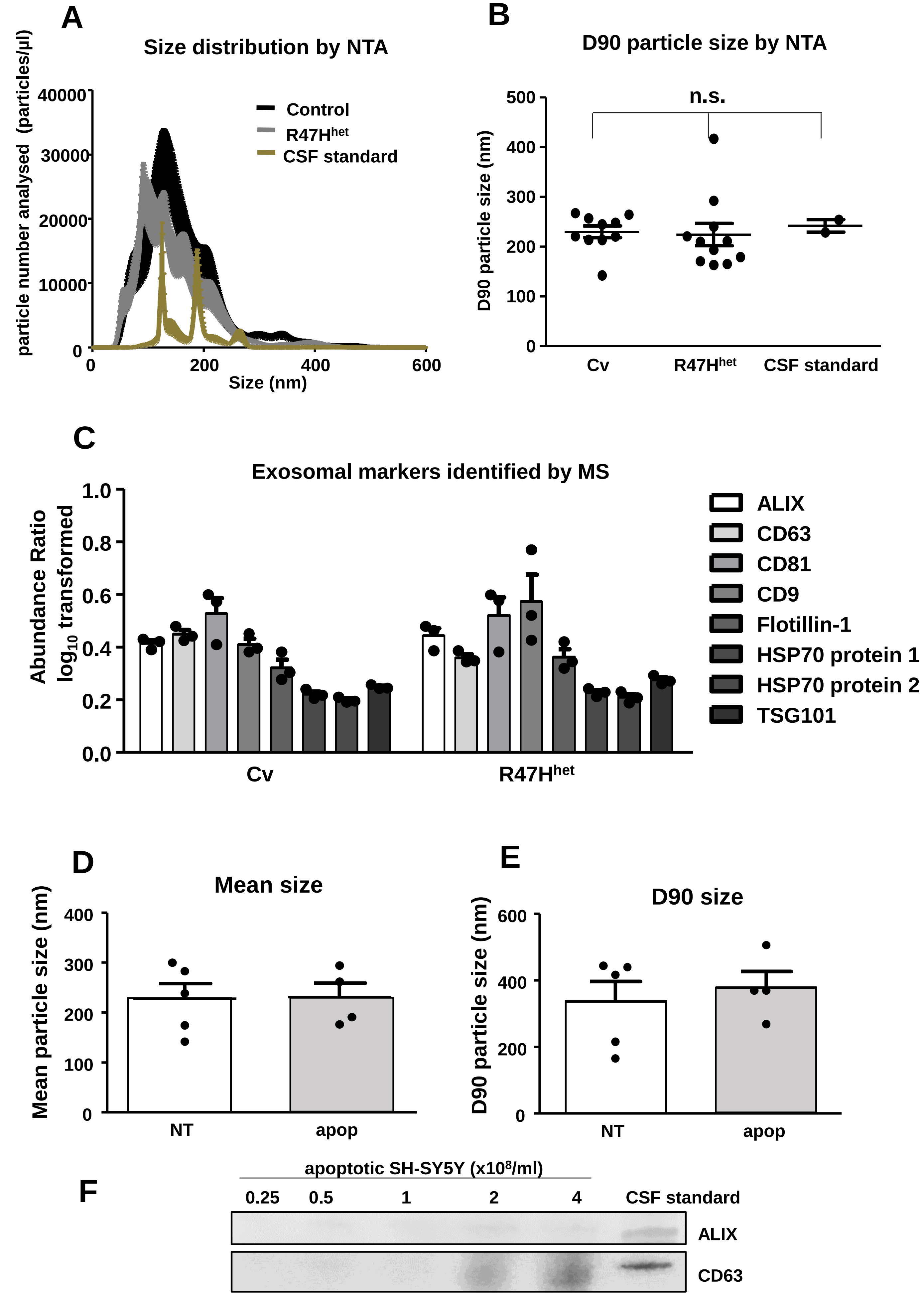

Supplementary Figure 1 –NTA and MS analyses of exosomes

(A) Nanoparticle tracking analysis (NTA) of size distribution of iPS-derived common variant and R47H^{net} exosomes compared with a human CSF exosome control standard including SEM omitted from Fig 1C for clarity.

(B) The sizes of D90 exosomes from Cv and R47H^{het} iPS-Mg was also assessed and, were similar to the mean size shown in Fig 1D, indicating no difference between Cv, R47H^{het} and the CSF standard. Expanded dataset can be found in Supplementary Fig 4. Data are the mean ±SEM. One-way ANOVA with n ≥ 4.

(C) Classical exosomal markers were also identified through LC-MS. Both Cv and R47H^{het} exosomes were shown to display a range of exosomal markers including ALIX, CD63, CD81, CD9. Data are the mean ±SEM. 2-way ANOVA with Turkey's post-hoc test with n = 3.

(D) To determine whether treatment of iPS-Mg with apoptotic neurons led to cross-contamination of iPSC-Mg exosomal fractions with apoptotic neuron-derived vesicles, such as apoptotic bodies, exosomal size was analysed before and after apoptotic neuron addition, as apoptotic bodies are larger in size than exosomes. The mean size of exosome particles analysed through NTA was not significantly different before and after apoptotic neuron treatment, suggesting no carry-over of potential apoptotic bodies.

(E) The D90 measurement of exosome particles analysed through NTA was also not significantly different before and after apoptotic neuron treatment.

(F) In addition to size analysis, the presence of exosomal markers in apoptotic bodies was also tested by western blot. Apoptotic SH-SY5Y neurons of varying amounts were extracted for exosomes and subjected to western blot with the exosomal marker ALIX, which was only detected in the CSF standard, The CD63 exosomal marker was detected at concentrations of apoptotic neurons above 2-4 $x10^6$ /ml. Based on this, a concentration of 10^6 apoptotic SH-SY5Y were used for all experiments. A, B: n = 10 for Cv, n = 13 for R47H^{het},C: n = 3, D, E: n = 4. B, F with one-way ANOVA and C with two-way ANOVA.

Supplementary Figure 2

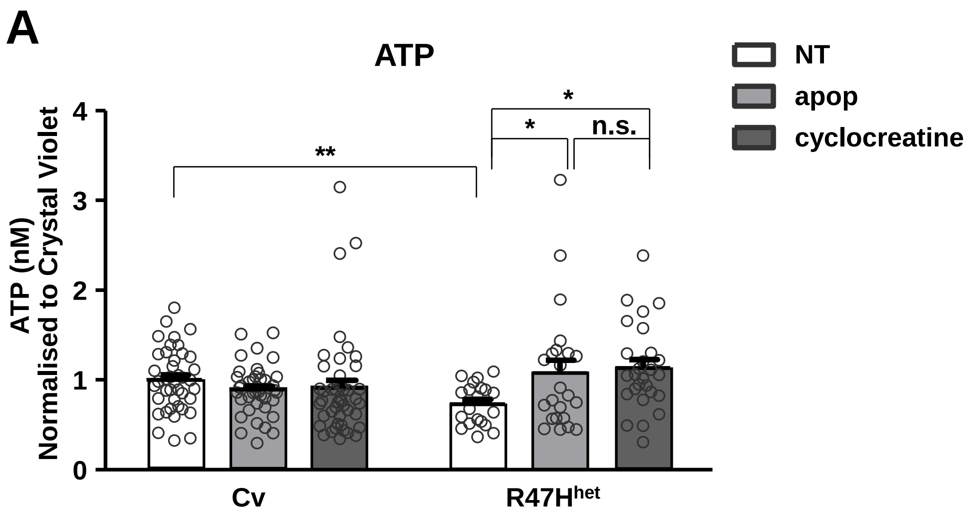

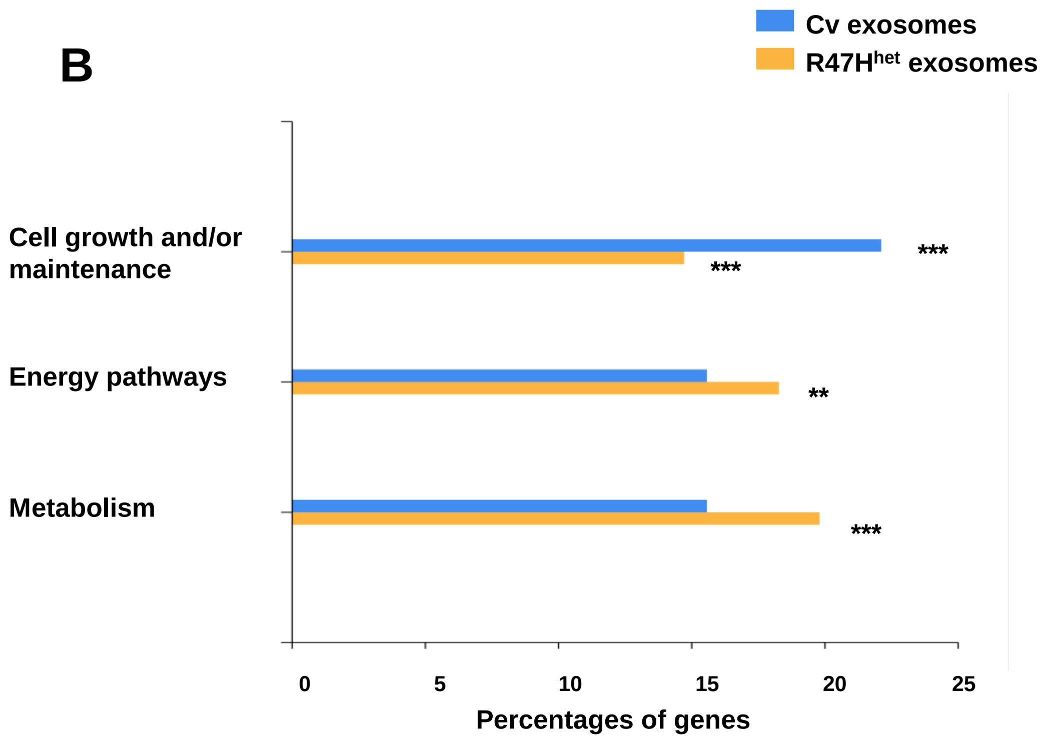

Supplementary Figure 2 continued

C

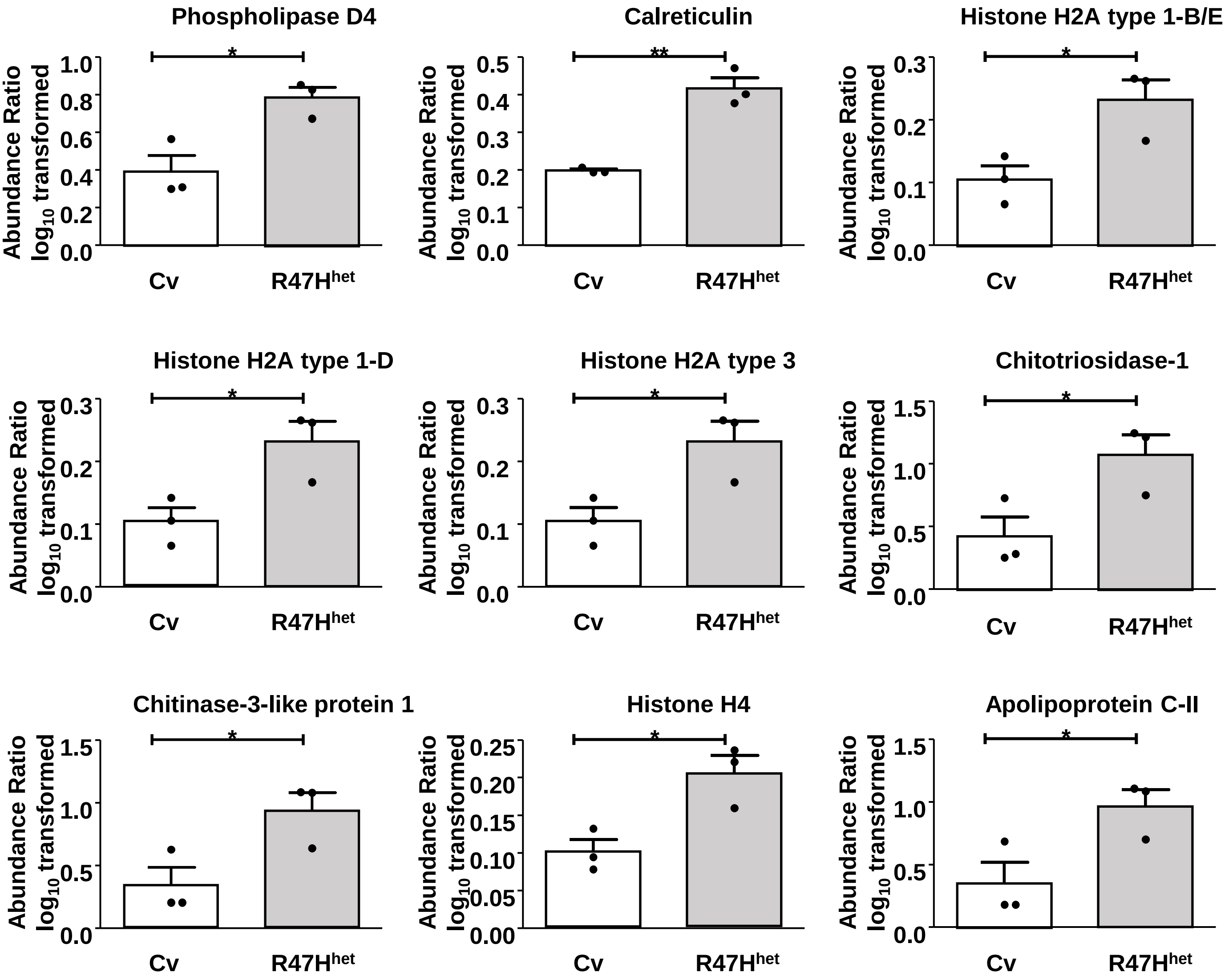

<u>**Supplementary Figure 2 –ATP availability and pathway analysis**</u>

(A) ATP availability was tested with a bioluminescence ATP assay and the results normalised to total protein concentration determined by BCA assay. At baseline, a reduced ATP availability was observed in the R47H^{het} iPS-Mg, which was rescued with treatment of iPS-Mg with apoptotic neurons (2:1 apoptotic neurons:iPS-Mg) or cyclocreatine (10 mM). . A: Two-way ANOVA with Tukey's post-hoc test with n=5 (for Cv) and n=3 (for R47H^{het}). * $\rho < 0.05$, ** $\rho < 0.01$, *** $\rho < 0.001$, n.s. not significant. Expanded dataset can be found in Supplementary Fig 4.

(B) Pathway analysis of the 200 most abundant proteins identified through LC-MS was performed using FunRich. Cv exosomes were enriched for proteins involved in cell growth and maintenance, whilst R47H^{het} exosomes were enriched for energy pathways and metabolism

(C) Differentially packaged proteins, highlighted **in Figure 3C**, plotted individually. Independent t-test with n=3 and * $\rho < 0.05$, ** $\rho < 0.01$.

Supplementary Figure 3

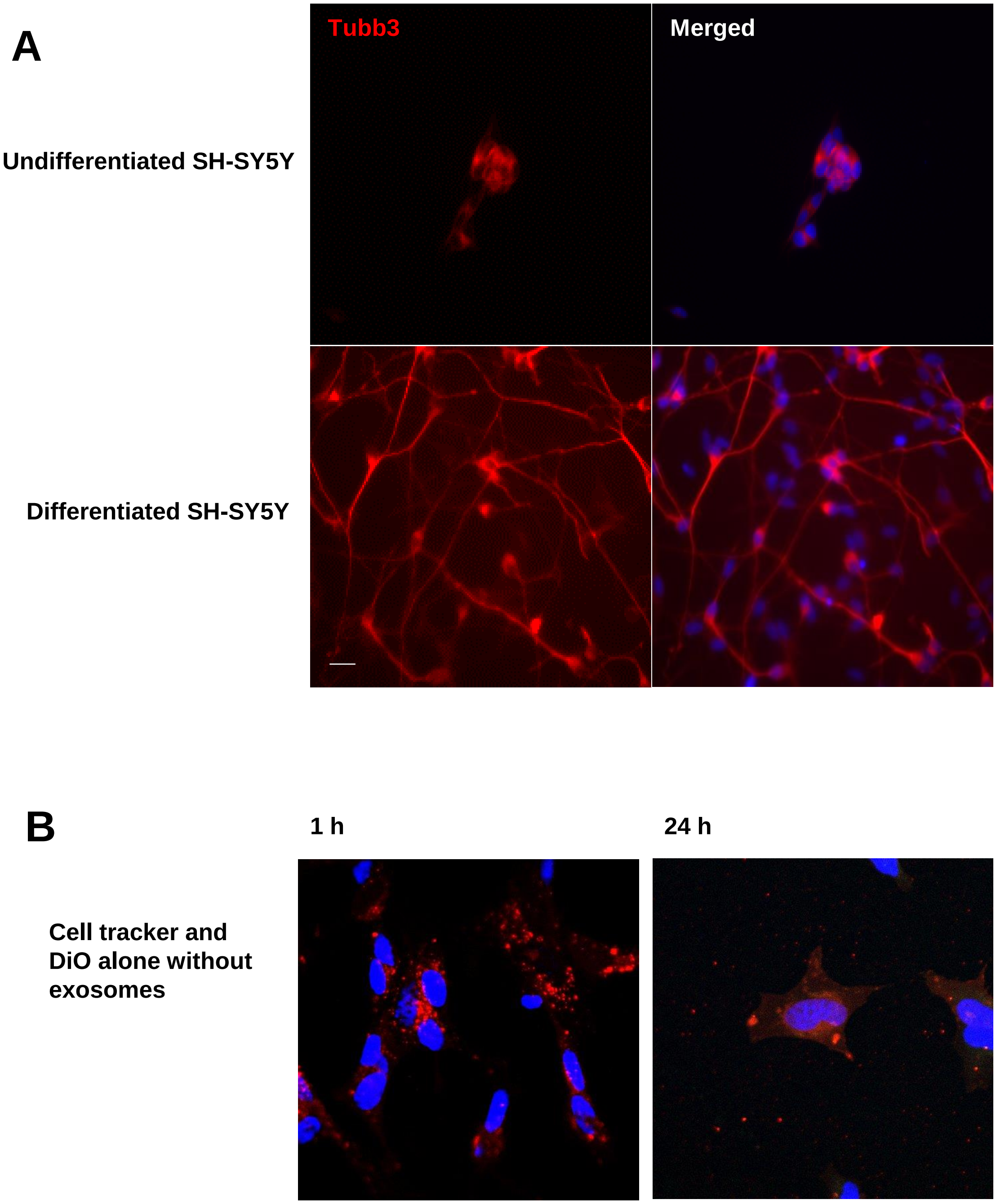

<u>Supplementary Figure 3 –SH-SY5Y differentiation and exosome tracking</u>

(A) Following an established protocol using retinoic acid and BNDF, SH-SY5Y cells were reliably differentiated out, leading to changes in morphology. TUBB3 staining. Scale bar = 10 µm.

(B) Tracking of DiO uptake without exosomes at 1 h or 24 h into SH-SY5Y neurons labelled with membrane marker Biotracker555 orange and the nuclear marker DAPI.

Supplementary Figure 4

Figure 1D

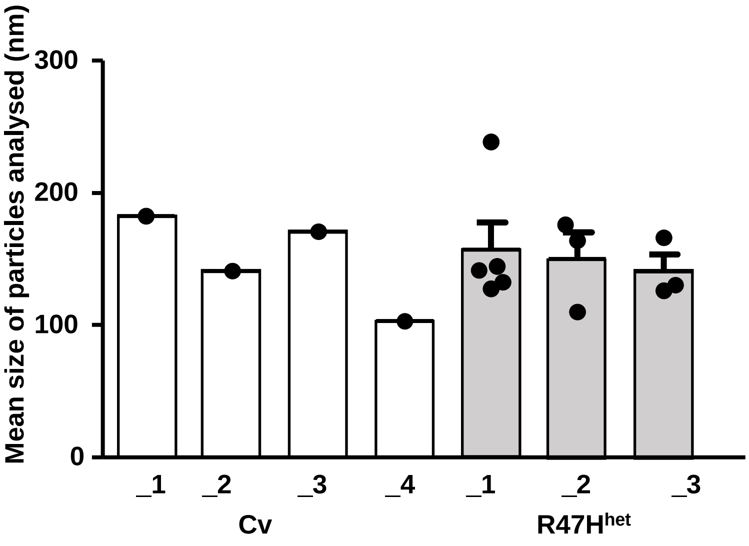

Figure 1E

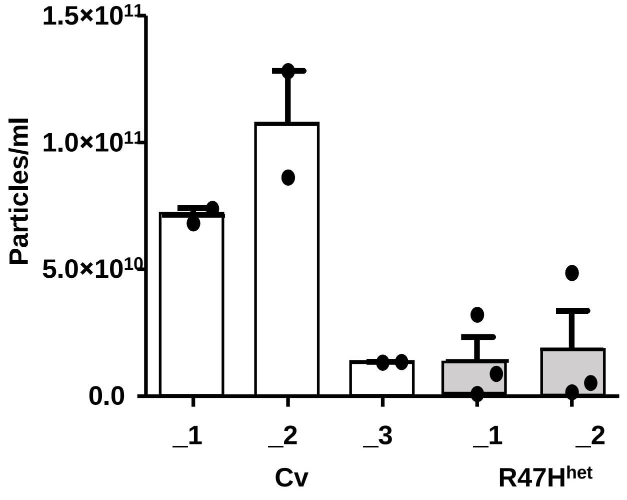

Figure 1F

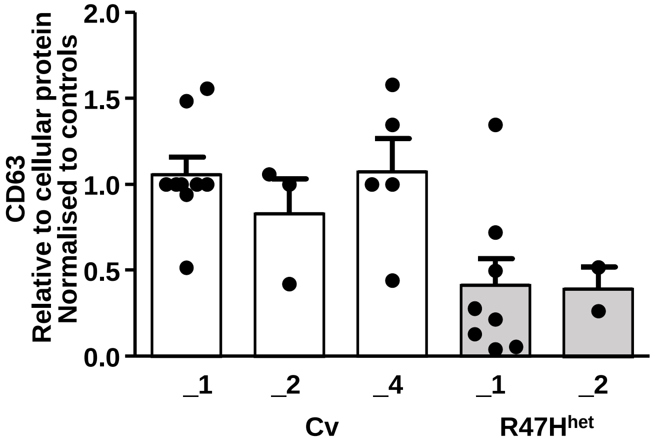

Figure 2B

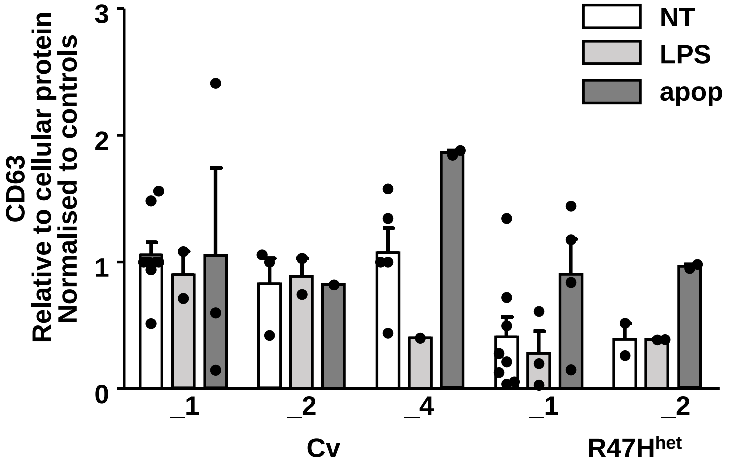

Figure 2D

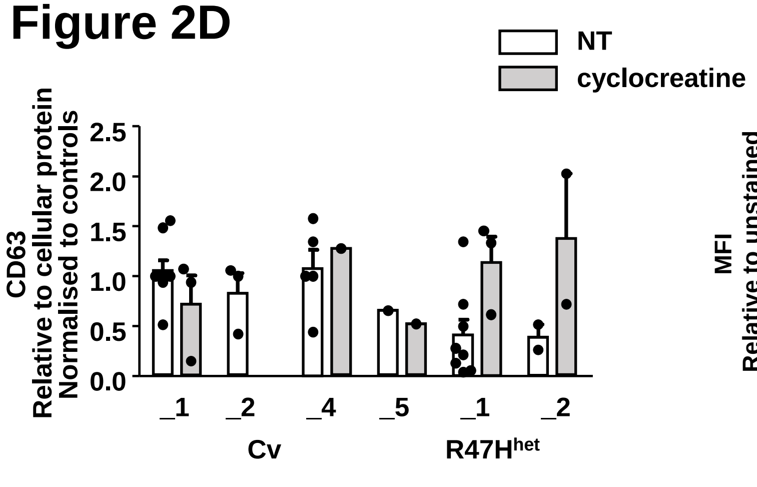

Figure 7D

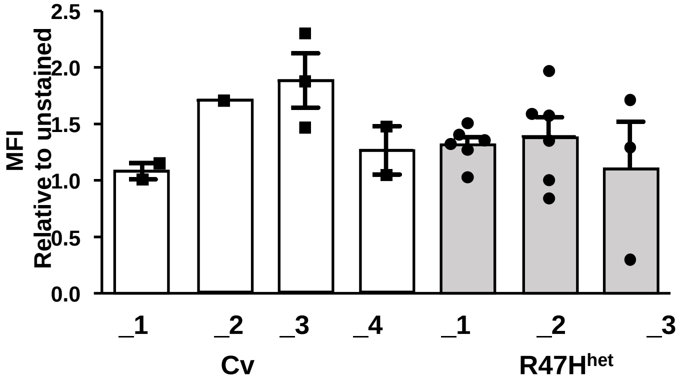

Figure 8B

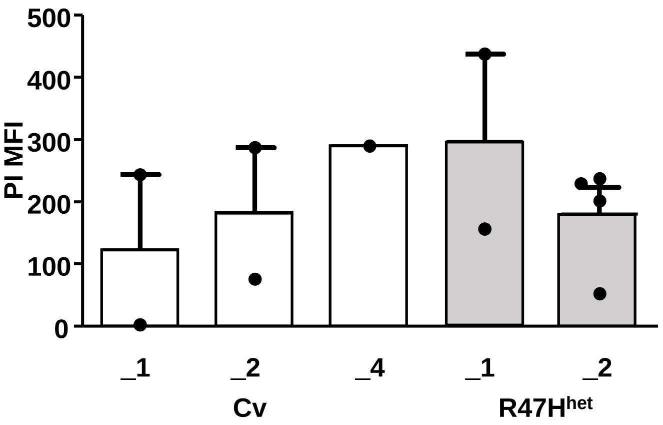

Figure 8D

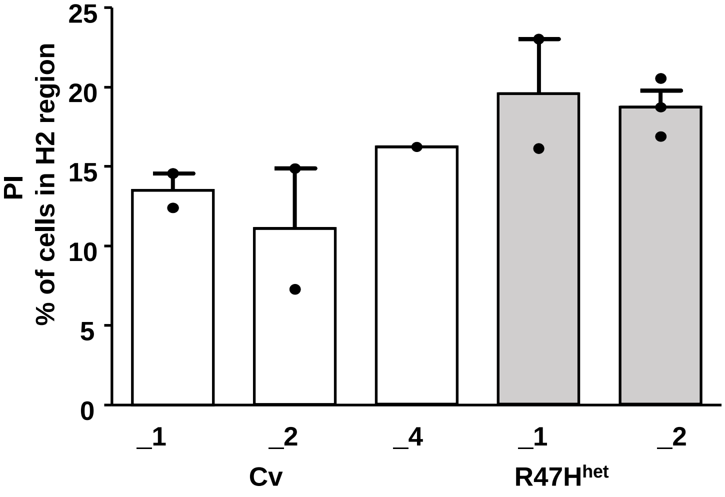

Supp Figure 1B

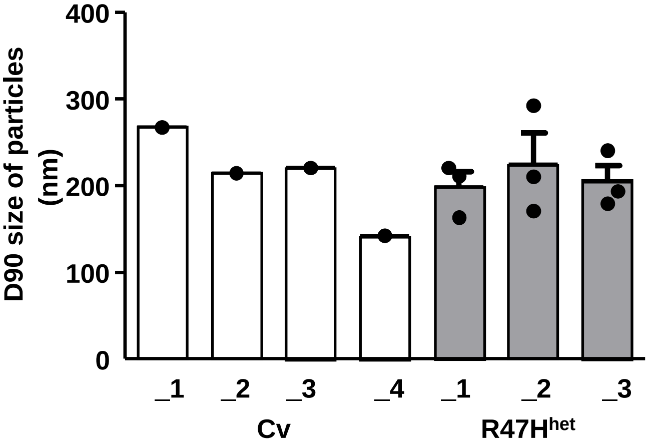

Supp Figure 2A

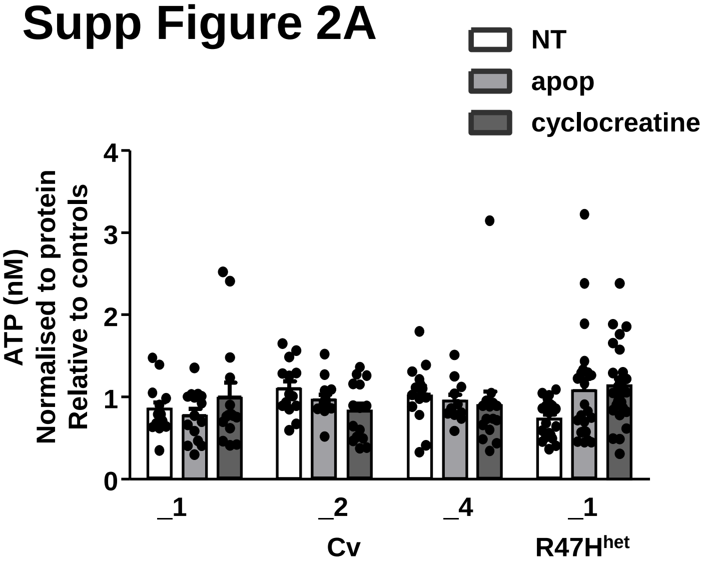

Supplementary Figure 4

Data corresponding to the main Figures (Figure 1D-F, Figure 2B, D, Figure 7D and Figure 8B and 8D) or Supplementary Figures (Supp Figure 1B and Supp Figure 2A) with all data points split according to cell or patient line

Supplementary Figure 5

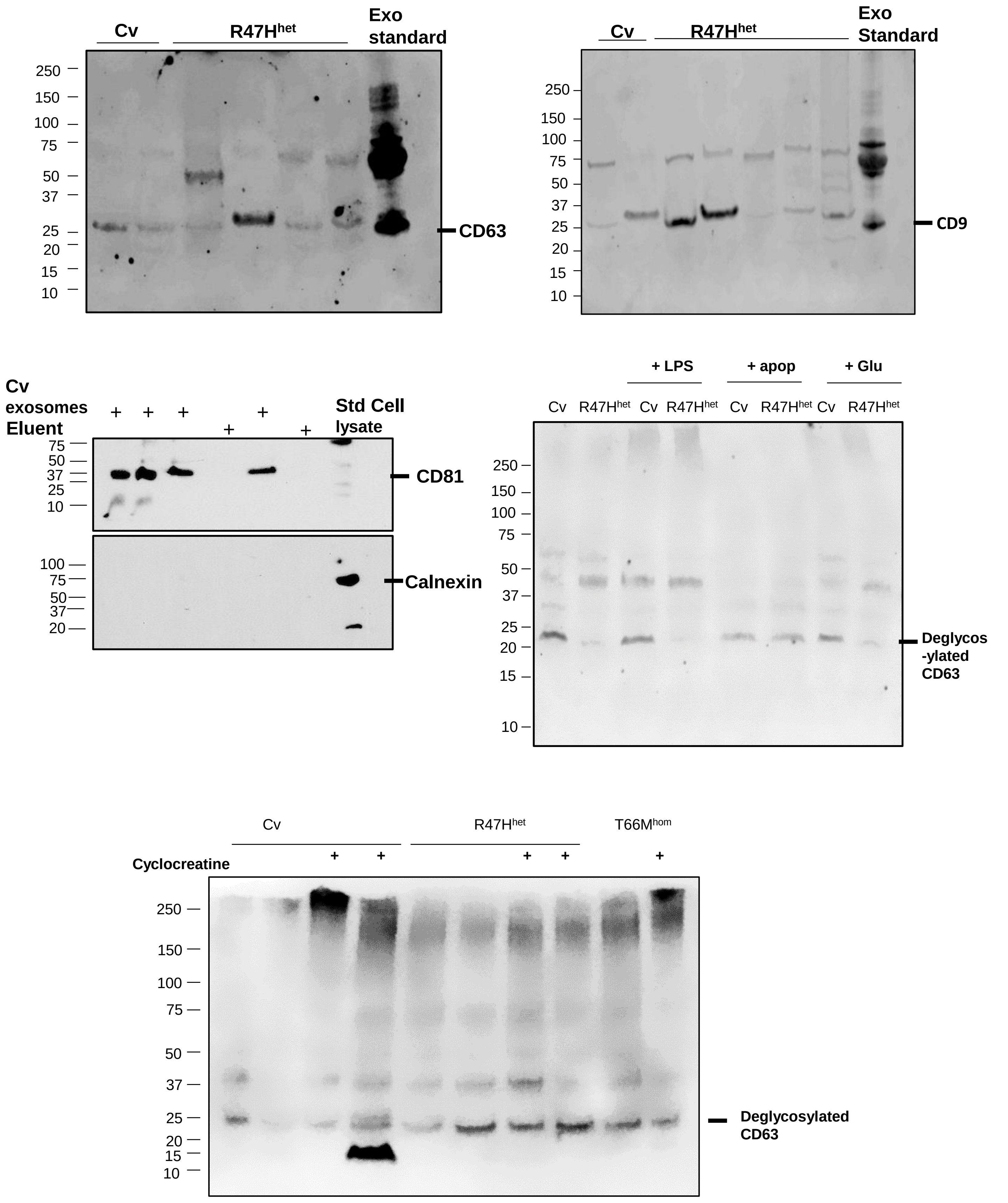

<u>Supplementary Figure 5–Uncropped Western Blots</u>

Uncropped western blots from **Figure 1** and **Figure 2.**
